# Flecainide Is Associated With a Lower Incidence of Arrhythmic Events in a Large Cohort of Patients With Catecholaminergic Polymorphic Ventricular Tachycardia

**DOI:** 10.1161/CIRCULATIONAHA.123.064786

**Published:** 2023-10-27

**Authors:** Auke T. Bergeman, Krystien V.V. Lieve, Dania Kallas, J. Martijn Bos, Ferran Rosés i Noguer, Isabelle Denjoy, Esther Zorio, Janneke A.E. Kammeraad, Puck J. Peltenburg, Katie Tobert, Takeshi Aiba, Joseph Atallah, Fabrizio Drago, Anjan S. Batra, Ramon Brugada, Martin Borggrefe, Sally-Ann B. Clur, Moniek G.P.J. Cox, Andrew Davis, Santokh Dhillon, Susan P. Etheridge, Peter Fischbach, Sonia Franciosi, Kristina Haugaa, Minoru Horie, Christopher Johnsrude, Austin M. Kane, Ulrich Krause, Sit-Yee Kwok, Martin J. LaPage, Seiko Ohno, Vincent Probst, Jason D. Roberts, Tomas Robyns, Frederic Sacher, Christopher Semsarian, Jonathan R. Skinner, Heikki Swan, Terezia Tavacova, Svjetlana Tisma-Dupanovic, Jacob Tfelt-Hansen, Sing-Chien Yap, Prince J. Kannankeril, Antoine Leenhardt, Janice Till, Shubhayan Sanatani, Michael W.T. Tanck, Michael J. Ackerman, Arthur A.M. Wilde, Christian van der Werf

**Affiliations:** 1 Heart Centre, Department of Cardiology (A.T.B., K.V.V.L., P.J.P., A.A.M.W., C.v.d.W.), Amsterdam UMC Location AMC, University of Amsterdam, The Netherlands.; 2 Department of Pediatric Cardiology, Emma Children’s Hospital (S.-A.B.C.), Amsterdam UMC Location AMC, University of Amsterdam, The Netherlands.; 3 Epidemiology and Data Science, Amsterdam Public Health, Methodology (M.W.T.T.), Amsterdam UMC Location AMC, University of Amsterdam, The Netherlands.; 4 Amsterdam Cardiovascular Sciences, Heart Failure and Arrhythmias, The Netherlands (A.T.B., K.V.V.L., P.J.P., A.A.M.W., C.v.d.W.).; 5 Department of Pediatrics, BC Children’s Hospital, University of British Columbia, Vancouver, Canada (D.K., S.F., S.S.).; 6 Departments of Cardiovascular Medicine, Pediatric and Adolescent Medicine, and Molecular Pharmacology & Experimental Therapeutics, Divisions of Heart Rhythm Services and Pediatric Cardiology, Windland Smith Rice Genetic Heart Rhythm Clinic and Windland Smith Rice Sudden Death Genomics Laboratory, Mayo Clinic, Rochester, MN (J.M.B., K.T., M.J.A.).; 7 Department of Cardiology, Royal Brompton Hospital, London, United Kingdom (F.R.y.N., J.T.).; 8 Department of Paediatric Cardiology, Vall d’Hebron University Hospital, Barcelona, Spain (F.R.y.N.).; 9 Service de Cardiologie et CRMR Maladies Cardiaques Héréditaires et Rares, APHP, Hôpital Bichat, Université Paris Cité, France (I.D., A.L.).; 10 Department of Cardiology, Hospital Universitario y Politécnico La Fe, Valencia, Spain (E.Z.).; 11 Unidad de Cardiopatías Familiares, Muerte Súbita y Mecanismos de Enfermedad, Instituto de Investigación Sanitaria La Fe, Valencia, Spain (E.Z.).; 12 Center for Biomedical Network Research on Cardiovascular Diseases, Madrid, Spain (E.Z.).; 13 Department of Pediatric Cardiology, Erasmus MC–Sophia, Rotterdam, The Netherlands (J.A.E.K.).; 14 Medical Genome Center, National Cerebral and Cardiovascular Center, Suita, Japan (T.A., S.O.).; 15 Department of Pediatrics, University of Alberta, Edmonton, Canada (J.A.).; 16 Department of Pediatric Cardiology and Cardiac Surgery, Bambino Gesù Children’s Hospital and Research Institute, Rome, Italy (F.D.).; 17 European Reference Network for Rare, Low Prevalence and Complex Diseases of the Heart: ERN GUARD-Heart (F.R.y.N., I.D., F.D., S.-A.B.C., V.P., T.R., F.S., H.S., T.T., J.T.-H., A.L., A.A.M.W., C.v.d.W.).; 18 Department of Pediatrics, University of California, Irvine (A.S.B.).; 19 Cardiovascular Genetics Center, Institut d’Investigació Biomèdica Girona, Hospital Trueta, CIBERCV, University of Girona, Spain (R.B.).; 20 Department of Medicine, University Medical Center Mannheim, Germany (M.B.).; 21 Department of Cardiology, University of Groningen, University Medical Centre Groningen, The Netherlands (M.G.P.J.C.).; 22 The Royal Children’s Hospital, Melbourne, Australia (A.D.).; 23 IWK Health Center, Dalhousie University, Halifax, Canada (S.D.).; 24 Division of Pediatric Cardiology, University of Utah, Salt Lake City (S.P.E.).; 25 Sibley Heart Center, Children’s Healthcare of Atlanta, GA (P.F.).; 26 ProCardio Center for Innovation, Heart, Vessel and Lung Clinic, Oslo University Hospital, Rikshospitalet, Norway (K.H.).; 27 Department of Cardiovascular Medicine, Shiga University of Medical Science, Otsu, Japan (M.H., S.O.).; 28 Division of Pediatric Cardiology, Department of Pediatrics, Norton Children’s Hospital, University of Louisville School of Medicine, KY (C.J.).; 29 University of Alabama at Birmingham (A.M.K.).; 30 Department of Pediatric Cardiology and Intensive Care Medicine, University Medical Center Göttingen, Georg-August-University, Germany (U.K.).; 31 Department of Paediatrics, Hong Kong Children’s Hospital, China (S.-Y.K.).; 32 University of Michigan Congenital Heart Center, Ann Arbor (M.J.L.).; 33 Université de Nantes, CHU Nantes, CNRS, INSERM, L’institut du Thorax, France (V.P.).; 34 Section of Cardiac Electrophysiology, Division of Cardiology, Department of Medicine, Western University, London, Canada (J.D.R.).; 35 Department of Cardiovascular Diseases, University Hospitals Leuven, Belgium (T.R.).; 36 LIRYC Institute, Bordeaux University Hospital, Bordeaux University, France (F.S.).; 37 Agnes Ginges Centre for Molecular Cardiology at Centenary Institute, University of Sydney, Australia (C.S.).; 38 Cardiac Inherited Disease Group New Zealand, Green Lane Paediatric and Congenital Cardiac Services, Starship Children’s Hospital, Auckland (J.R.S.).; 39 Heart and Lung Centre, Helsinki University Hospital and Helsinki University, Finland (H.S.).; 40 Children’s Heart Centre, 2nd Faculty of Medicine, Charles University in Prague and Motol University Hospital, Czech Republic (T.T.).; 41 Division of Cardiology, Children’s Mercy Hospital, Kansas City, MO (S.T.-D.).; 42 Department of Cardiology, Rigshospitalet, Copenhagen, Denmark (J.T.-H.).; 43 Section of Genetics, Department of Forensic Medicine, Faculty of Medical Sciences, University of Copenhagen, Denmark (J.T.-H.).; 44 Department of Cardiology, Erasmus MC, University Medical Center Rotterdam, The Netherlands (S.-C.Y.).; 45 Department of Pediatrics, Monroe Carell Jr Children’s Hospital at Vanderbilt, Vanderbilt University Medical Centre, Nashville, TN (P.J.K.).

**Keywords:** catecholaminergic polymorphic ventricular tachycardia, sudden cardiac death, ventricular arrhythmias

## Abstract

**BACKGROUND:**

In severely affected patients with catecholaminergic polymorphic ventricular tachycardia, beta-blockers are often insufficiently protective. The purpose of this study was to evaluate whether flecainide is associated with a lower incidence of arrhythmic events (AEs) when added to beta-blockers in a large cohort of patients with catecholaminergic polymorphic ventricular tachycardia.

**METHODS:**

From 2 international registries, this multicenter case cross-over study included patients with a clinical or genetic diagnosis of catecholaminergic polymorphic ventricular tachycardia in whom flecainide was added to beta-blocker therapy. The study period was defined as the period in which background therapy (ie, beta-blocker type [beta1-selective or nonselective]), left cardiac sympathetic denervation, and implantable cardioverter defibrillator treatment status, remained unchanged within individual patients and was divided into pre-flecainide and on-flecainide periods. The primary end point was AEs, defined as sudden cardiac death, sudden cardiac arrest, appropriate implantable cardioverter defibrillator shock, and arrhythmic syncope. The association of flecainide with AE rates was assessed using a generalized linear mixed model assuming negative binomial distribution and random effects for patients.

**RESULTS:**

A total of 247 patients (123 [50%] females; median age at start of flecainide, 18 years [interquartile range, 14–29]; median flecainide dose, 2.2 mg/kg per day [interquartile range, 1.7–3.1]) were included. At baseline, all patients used a beta-blocker, 70 (28%) had an implantable cardioverter defibrillator, and 21 (9%) had a left cardiac sympathetic denervation. During a median pre-flecainide follow-up of 2.1 years (interquartile range, 0.4–7.2), 41 patients (17%) experienced 58 AEs (annual event rate, 5.6%). During a median on-flecainide follow-up of 2.9 years (interquartile range, 1.0–6.0), 23 patients (9%) experienced 38 AEs (annual event rate, 4.0%). There were significantly fewer AEs after initiation of flecainide (incidence rate ratio, 0.55 [95% CI, 0.38–0.83]; *P*=0.007). Among patients who were symptomatic before diagnosis or during the pre-flecainide period (n=167), flecainide was associated with significantly fewer AEs (incidence rate ratio, 0.49 [95% CI, 0.31–0.77]; *P*=0.002). Among patients with ≥1 AE on beta-blocker therapy (n=41), adding flecainide was also associated with significantly fewer AEs (incidence rate ratio, 0.25 [95% CI, 0.14–0.45]; *P*<0.001).

**CONCLUSIONS:**

For patients with catecholaminergic polymorphic ventricular tachycardia, adding flecainide to beta-blocker therapy was associated with a lower incidence of AEs in the overall cohort, in symptomatic patients, and particularly in patients with breakthrough AEs while on beta-blocker therapy.

**Clinical Perspective**

**What Is New?**

- In catecholaminergic polymorphic ventricular tachycardia, addition of flecainide to beta-blocker therapy is associated with a reduction in arrhythmic events, defined as syncope, appropriate implantable cardioverter defibrillator shock, sudden cardiac arrest, or sudden cardiac death, compared with beta-blocker monotherapy.
- Patients who remained symptomatic despite beta-blocker therapy had the greatest reduction in arrhythmic events after the addition of flecainide.

**What Are the Clinical Implications?**

- In patients with catecholaminergic polymorphic ventricular tachycardia and incomplete control of ventricular arrhythmias, flecainide should be added to their therapeutic regimen.
- Clinicians should consider initiating dual therapy of a beta-blocker and flecainide immediately rather than beta-blocker monotherapy in high-risk patients.

Catecholaminergic polymorphic ventricular tachycardia (CPVT) is a rare and potentially lethal inherited cardiac arrhythmia syndrome caused by abnormal intracellular calcium homeostasis of cardiac cells. Characteristically, polymorphic or bidirectional ventricular arrhythmias (VAs) occur under circumstances of high sympathetic tone, potentially inducing syncope, sudden cardiac arrest (SCA), and sudden cardiac death (SCD).^1^

Since the earliest clinical reports, beta-blockers have been the cornerstone of therapy for preventing arrhythmic events (AEs) in CPVT, and current guidelines advocate treating every clinically diagnosed patient with CPVT with a beta-blocker, preferably a nonselective beta-blocker.^2^ However, some patients are insufficiently protected by beta-blockers alone, and severe VAs or breakthrough AEs on beta-blocker therapy occur.^3,4^ In addition, beta-blockers may cause side effects in some patients, leading to nonadherence or discontinuation of therapy.^5^

Flecainide is a class IC antiarrhythmic drug that is effective in suppressing VAs in patients with CPVT,^6,7^ and guidelines state that flecainide should be considered as the first addition to beta-blockers when control of VAs or AEs is incomplete.^2^ However, current evidence for the efficacy of flecainide in reducing AEs in patients with CPVT relies on relatively small cohorts with short-term follow-up.^5,7–10^ The purpose of this study was to assess the association of flecainide with the incidence of AEs in a large cohort of patients with CPVT from 2 international CPVT registries with long-term follow-up.

## METHODS

### Study Design, Setting, and Population

The study sample was derived from the International CPVT Registry and the International Pediatric CPVT Registry. The International CPVT Registry is a retrospective, international, multicenter registry of patients with CPVT instituted in 2014 that included patients from 37 center at the time of this study (March 2022). The International Pediatric CPVT Registry is an international multicenter registry of pediatric patients with CPVT and included patients from 27 centers, primarily from the Pediatric and Congenital Electrophysiology Society, at the time of this study. Both the International CPVT Registry and the International Pediatric CPVT Registry were initiated as retrospective cohort studies, but follow-up information has been collected prospectively. Appropriate research ethics board approval and informed consent have been obtained at all participating centers. The data that support the findings of this study are available from the corresponding author upon reasonable request.

For this study, we included all patients with CPVT who were treated for at least 7 days with a beta-blocker, either as monotherapy or combined with left cardiac sympathetic denervation (LCSD), and in whom flecainide was subsequently added to beta-blocker therapy. The indication for adding flecainide was at the discretion of the treating physician. Patients with and without implantable cardioverter defibrillators (ICDs) were included.

Patients <65 years of age upon initiation of flecainide were included. Patients who did not have a (likely) pathogenic variant in ryanodine receptor 2 (*RYR2*) or homozygous or compound heterozygous variants in *CASQ2* were included only if a definite CPVT phenotype was present.^11–13^ This was defined as bigeminal ventricular premature beats or more complex VAs on exercise stress test, epinephrine challenge test, or Holter monitoring.

We excluded patients with a *RYR2* exon 3 deletion,^14,15^ those with a known *RYR2* loss-of-function variant,^16,17^ and those with a (likely) pathogenic variant in *KCNJ2*. In addition, patients with significant structural heart disease, defined as current or a history of left ventricular ejection fraction <40% or significant coronary artery stenosis, moderate to severe valvular stenosis or regurgitation, evidence of cardiomyopathy on echocardiography or cardiac magnetic resonance imaging, or congenital heart disease with significant hemodynamic consequences, were excluded.

### Outcome

The primary outcome of this study was the number of AEs, which were defined as a composite of SCD, SCA, appropriate ICD shock, or arrhythmic syncope/seizures of (presumed) cardiac cause.

### Follow-Up

Two study periods were defined. The first study period was the “pre-flecainide period” and was defined as the period after diagnosis during which a beta-blocker was prescribed until initiation of flecainide; the second study period was referred to as the “on-flecainide period,” defined as the period after diagnosis in which combination therapy of beta-blocker and flecainide was used. It is important to note that background therapy, defined as beta-blocker type (selective or nonselective),^18^ performance of LCSD, and presence or absence of an ICD, had to be unchanged during the pre- and on-flecainide study periods. For example, if a patient experienced an AE on beta-blocker therapy and an ICD was inserted while flecainide was initiated, the patient was not included. The rationale for this choice is that the presence of an ICD may influence event rates, because ICD carriers sometimes experience shocks secondary to VAs, which would not have caused syncope or SCA in the absence of an ICD.^19^ For patients who switched beta-blockers within a group (selective or nonselective) between the study periods (eg, metoprolol to atenolol), dosage equivalence was checked using the dose equivalence table by Roston et al (Table S1).^20^ The pre-flecainide period started when the background therapy that was present in the on-flecainide period was initiated. The follow-up of the on-flecainide period was censored on the date of last follow-up or when a change in background therapy occurred. The rationale behind this was to enable estimation of the unbiased effect of flecainide on top of stable background therapy.

Two secondary analyses in which patients who received an ICD when flecainide was initiated or in whom the date of ICD insertion during the pre-flecainide or on-flecainide periods was not used as the starting date or date of censoring, respectively, are detailed in the Supplemental Material.

To account for varying observation times in the 2 study periods, the incidence rate of AEs during the pre-flecainide and on-flecainide periods was calculated by dividing the total number of AEs by the total follow-up duration of all patients and expressed as the average number of AEs per patient-year. On the basis of the intention-to-treat principle, AEs occurring during periods of nonadherence were included.

Patients were divided into 3 categories: (1) all patients; (2) all symptomatic patients (defined as those with a history of syncope or SCA before flecainide therapy was initiated); and (3) beta-blocker–resistant patients who experienced ≥1 AE despite beta-blocker therapy during the pre-flecainide period.

### Statistical Analysis

Continuous variables were expressed as median with interquartile range (IQR) and compared with the Wilcoxon signed-rank test, or Mann-Whitney *U* test when appropriate. Categorical variables were reported as absolute and relative frequencies and compared by Fisher exact or McNemar test as appropriate. To assess the effect of flecainide on the rate of AEs, a generalized linear mixed model assuming a negative binomial distribution for the AE counts, a random effect for patients, and correction for cohort (International CPVT Registry versus International Pediatric CPVT Registry) and the length of the pre-flecainide and on-flecainide follow-up were used. The length of the respective off- and on-flecainide periods and the cohort of the patient were included as covariates in the negative binomial generalized linear mixed models. Data were analyzed using R version 4.1.3 (The R Project for Statistical Computing). A 2-tailed *P* value ≤0.05 was considered statistically significant. One author (A.T.B.) had full access to the data of both registries and takes responsibility for the data integrity and analysis.

## RESULTS

### Patient Characteristics

**Figure 1.**
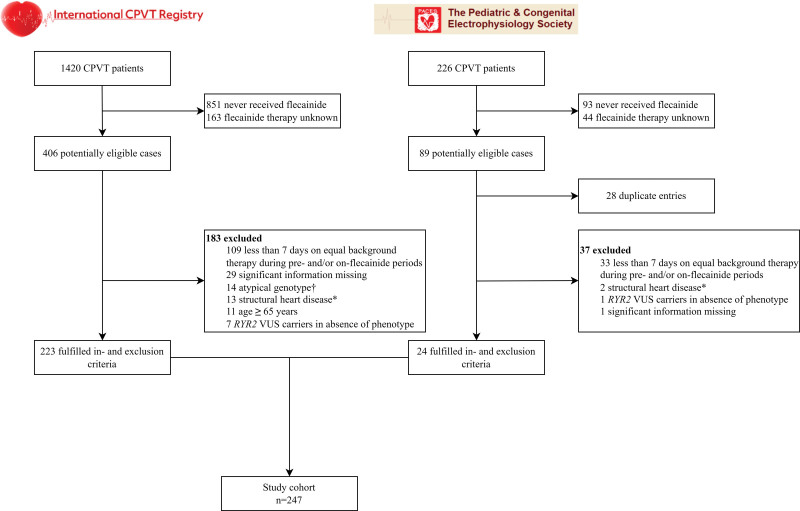
**Flowchart of study participants.** *Defined as current or a history of left ventricular ejection fraction <40% or significant coronary artery stenosis, moderate to severe valvular stenosis or regurgitation, evidence of cardiomyopathy on echocardiography or cardiac magnetic resonance imaging, or congenital heart disease with significant hemodynamic consequences. †Defined as *RYR2* exon 3 deletion, a *RYR2* loss-of-function variant, or a (likely) pathogenic variant in *KCNJ2*. CPVT indicates catecholaminergic polymorphic ventricular tachycardia; *RYR2*, ryanodine receptor 2; and VUS, variant of unknown significance.

A total of 247 patients with CPVT (123 [50%] female; median age at diagnosis, 13 years [IQR, 9–22]) who were diagnosed with CPVT between 1980 and 2020 were included (Figure 1; Table 1). Before diagnosis, 164 (66%) patients were symptomatic. Forty-one (17%) patients were symptomatic after the initiation of beta-blocker therapy, including 3 patients who experienced their first symptom on beta-blocker monotherapy. The majority of patients (88%) had *RYR2*-mediated CPVT, including 19 *RYR2*-R420W variant-positive subjects. The characteristics of the patients with CPVT who were not treated with flecainide are shown in Table S2.

**Table 1.**
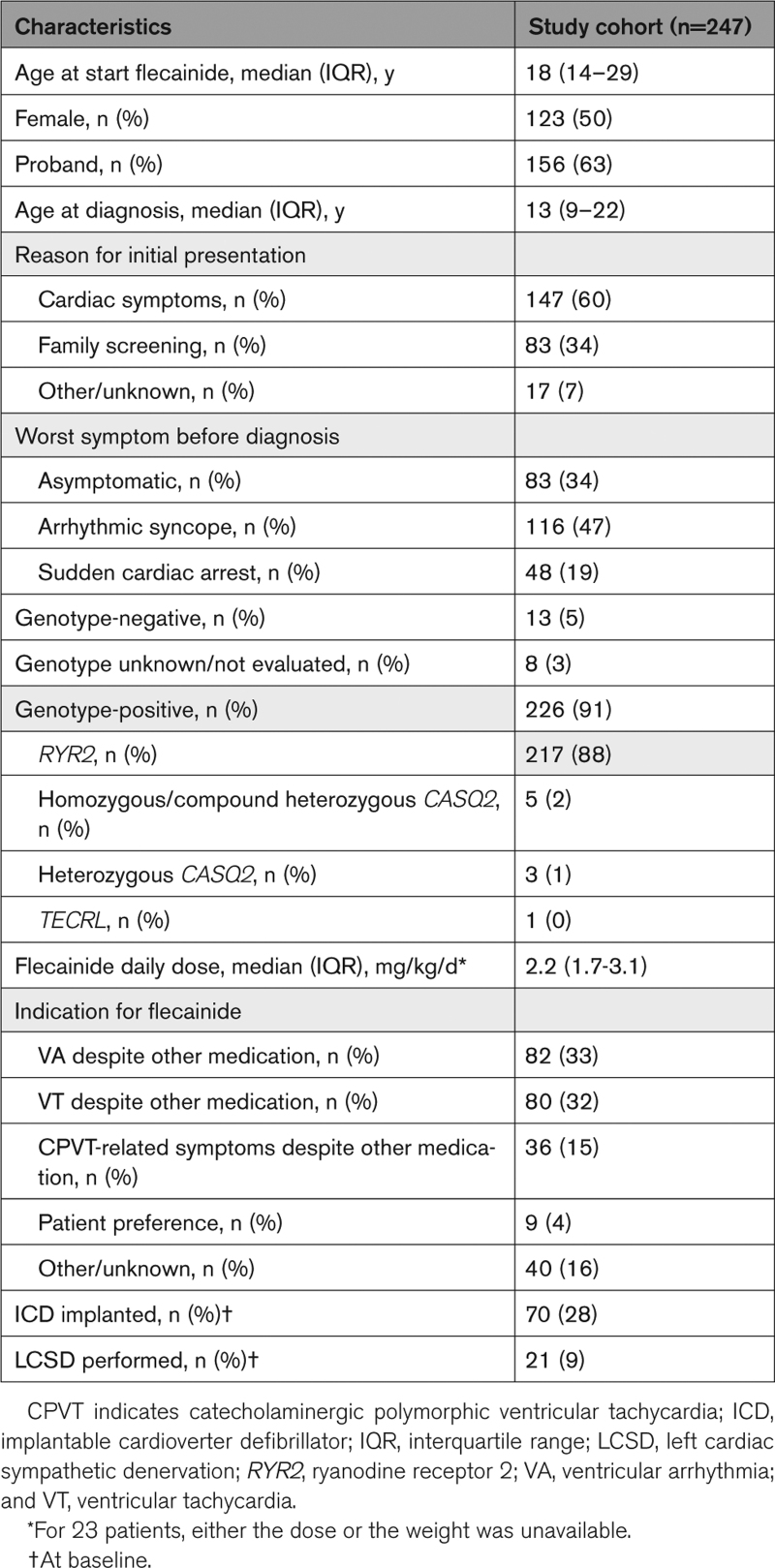
Clinical Characteristics of the Study Cohort

Patients were prescribed a median flecainide dose of 2.2 mg/kg per day (IQR, 1.7–3.1). For patients 18 years of age or older for whom the milligrams per day flecainide dose was available (n=98), the median flecainide dose was 150 mg per day (IQR, 100–200). During the study period, flecainide was stopped in 13 patients. The reasons for discontinuation were side effects in 5 patients, nonadherence in one patient, and unknown in the remaining 7 patients.

Beta-blocker types and doses that patients used during the study periods are shown in Table 2. Nadolol was the most prescribed beta-blocker during both the pre-flecainide period and the on-flecainide period. Despite the fact that 26 patients (11%) used a different beta-blocker dose (including 16 patients who received a lower beta-blocker dose) during the on-flecainide period compared with the pre-flecainide period, the median beta-blocker dosages were similar in both study periods (Table 2). In 18 patients, the beta-blocker dose equivalence could not be checked because of an unknown dosage in either study period. There were 34 patients who switched beta-blocker within the selective or nonselective group between the study periods.

**Table 2.**
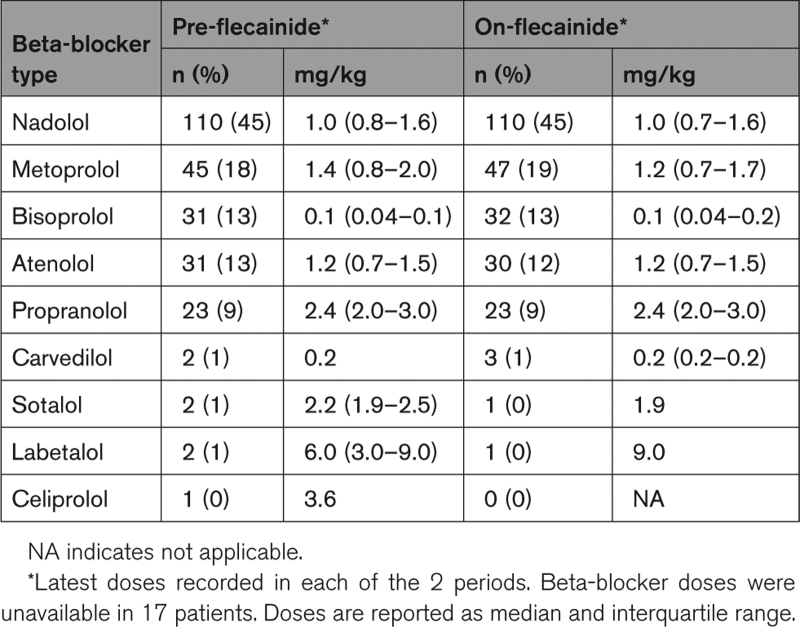
Types and Doses of Beta-Blockers During the Pre-Flecainide and On-Flecainide Periods

### Outcomes

**Figure 2.**
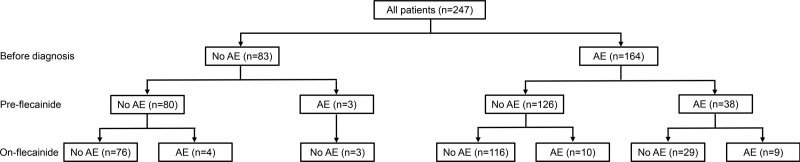
**Flowchart of the study cohort showing the number of patients with arrhythmic events before diagnosis and during the pre-flecainide and on-flecainide periods.** AE indicates arrhythmic event.

**Figure 3.**
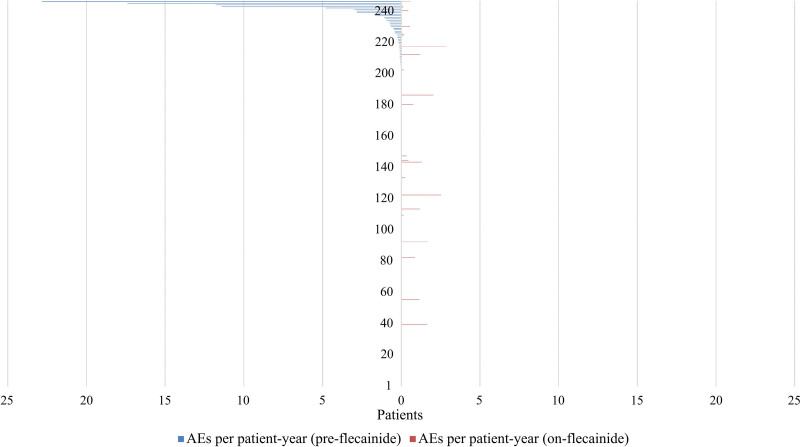
**Distribution of arrhythmic events per patient-year during the study period. Left**, pre-flecainide period and the on-flecainide period (**right**). The number of arrhythmic events per patient-year during the pre-flecainide and on-flecainide periods for 247 patients. Each line on either side of the vertical line (initiation of flecainide) represents one patient and the corresponding arrhythmic event rate per patient-year. AE indicates arrhythmic event.

The on-flecainide outcomes according to AEs before diagnosis and AEs during the pre-flecainide period are summarized in Figure 2. In the overall cohort (first group, 247 patients), the median duration of pre-flecainide follow-up was 2.1 years (IQR, 0.4–7.2). During the pre-flecainide period, 41 patients on beta-blocker monotherapy (17%) experienced a total of 58 AEs (4 [7%] SCA, 34 [59%] appropriate ICD shocks, 20 [34%] syncope), corresponding to an annual event rate of 5.6 per 100 patient-years (Figure 3). Of these patients, 21 (51%) used a nonselective beta-blocker, and 5 (12%) had undergone LCSD. Nine patients had multiple AEs during the pre-flecainide period: 5 patients had 2 AEs, 1 patient had 3 AEs, 2 patients had 4 AEs, and 1 patient had 5 AEs. The AE rate was higher in the pre-flecainide period compared with the post-flecainide period in all 9 patients.

The median duration of on-flecainide follow-up was 2.9 years (IQR, 1.0–6.0). During this period, 23 patients (9%) experienced a total of 38 AEs (1 [3%] SCD, 5 [13%] SCA, 16 [42%] appropriate ICD shocks, 16 syncope [42%]), corresponding to an event rate of 4.0 per 100 patient-years (Figure 3). Of these patients, 13 (57%) used a nonselective beta-blocker, and 2 (9%) had undergone LCSD. The median flecainide dose prescribed at the time of the AEs during the on-flecainide period was 2.5 mg/kg per day (IQR, 1.9–4.0; unknown in 5 patients). Five patients had multiple AEs during the post-flecainide period: 1 patient had 2 AEs, 2 patients had 3 AEs, 1 patient had 4 AEs, and 1 patient had 8 AEs. The AE rate was higher in the pre-flecainide period compared with the post-flecainide period in 2 out of 5 patients.

Among the 23 patients who experienced an AE on flecainide, 7 (30%) were either nonadherent or received a low dose (<100 mg daily) at the time of all AEs. This included a patient who died suddenly while on combination therapy with beta-blocker and flecainide. He was a 22-year-old-man and had fatal cardiac arrest while playing football. Six months before, he experienced syncope during exercise on nadolol monotherapy, prompting the addition of flecainide. He was adherent with the prescribed beta-blocker, but was nonadherent with respect to flecainide at the time of his death. He did not have an ICD implanted. The 16 patients (70%) who experienced at least 1 AE on an optimal dosage of flecainide and in whom nonadherence was not confirmed were generally severely affected patients. These patients experienced symptom onset at a median age of 9 years, nearly half had SCA before diagnosis, and 8 (50%) had a history of intellectual disability or neurodevelopmental disorder (Table S3). Four patients who were asymptomatic before diagnosis and during the pre-flecainide period experienced their first AE on flecainide. In all 4 patients, flecainide had been added because of clear worsening of the phenotype including significant VAs at exercise stress testing. In 2 of the patients, the AE was associated with nonadherence.

Overall, there was a significantly lower incidence of AEs after the initiation of flecainide (incidence rate ratio, 0.55 [95% CI, 0.38–0.85]; *P*=0.007).

In the second group, ie, the subgroup of patients who were symptomatic before diagnosis or on beta-blocker therapy (n=167), the median pre-flecainide follow-up duration was 2.1 years (IQR, 0.4–6.1), during which 41 patients experienced 58 AEs (4 [7%] SCA, 34 [59%] appropriate ICD shocks, 20 [34%] syncope), corresponding to an event rate of 7.8 per 100 patient-years. The median on-flecainide follow-up duration was 2.5 years (IQR, 0.9–5.5), during which 19 patients experienced 34 AEs (1 [3%] SCD, 3 [9%] SCA, 16 [47%] appropriate ICD shocks, 14 [41%] syncope), corresponding to an event rate of 5.6 per 100 patient-years, constituting a significantly lower incidence of AEs (incidence rate ratio, 0.48 [95% CI, 0.31–0.77]; *P*=0.002).

In the third and most severely affected subgroup, ie, the 41 patients who continued to have AEs despite beta-blocker therapy, the median pre-flecainide follow-up duration was 5.4 years (IQR, 1.2–9.3), during which the 41 patients experienced 58 AEs (4 [7%] SCA, 34 [59%] appropriate ICD shocks, 20 [34%] syncope), corresponding to an event rate of 24.8 per 100 patient-years. The median on-flecainide follow-up duration was 2.3 years (IQR, 0.8–6.2), during which 9 patients experienced 14 AEs (1 [7%] SCD, 2 [14%] SCA, 4 [29%] appropriate ICD shocks, 7 [50%] syncope), corresponding to an event rate of 9.5 per 100 patient-years. This constituted in a very significantly lower incidence of AEs (incidence rate ratio, 0.25 [95% CI, 0.14–0.45]; *P*<0.001).

## DISCUSSION

Our findings, derived from the largest cohort of patients with CPVT treated with flecainide, provide further evidence that flecainide has an important role in the treatment of CPVT. We demonstrated that the addition of flecainide to beta-blocker therapy is associated with a significantly lower incidence of AEs both in the overall cohort as well as in symptomatic patients, and especially in patients who experience breakthrough AEs while on beta-blocker therapy.

### Flecainide in Patients With CPVT

Flecainide is a class IC antiarrhythmic drug that has been used for the management of arrhythmias for decades. The efficacy of flecainide in CPVT was first suggested in 2009, when it was shown that flecainide dramatically reduced rates of calcium sparks in vitro, as well as VAs in vivo, in a *CASQ2*-knockout mouse model and in 2 patients.^21^ This finding led to subsequent studies showing a reduction in VA burden on exercise stress tests in patients with CPVT when treated with flecainide in addition to beta-blockers.^5,7–10^ Furthermore, flecainide was effective in patients with CPVT carrying homozygous *CASQ2* variants.^10^ Previous studies have suggested that flecainide use, particularly combined with beta-blocker therapy, results in a reduction in AEs.^22^ However, to date, this has not been analyzed systematically in a robust study with a significant number of patients and follow-up duration.

More recently, Kannankeril et al showed, in a randomized placebo-controlled cross-over trial, that flecainide in combination with beta-blockers was superior in reducing VAs during exercise stress tests in patients with CPVT compared with beta-blockers alone.^6^ In this trial, the initial primary end point of AEs was changed to VA burden on exercise stress test after recruiting a sufficient number of patients proved insurmountable. It is important to note that none of the patients in this trial had complex VAs (couplets or nonsustained VTs) on the exercise stress test while using the combination of beta-blockers and flecainide. The presence of couplets and the presence of nonsustained VTs are considered risk factors for AEs during follow-up.^23^

Our study adds to these previous observations, showing that flecainide is associated with a significantly lower incidence of AEs when added to beta-blocker therapy in the setting of no other therapeutic changes. Because no changes in background therapy were allowed during the pre-flecainide and on-flecainide study periods (including a switch from nonselective to beta1-selective beta-blockers or vice versa), it is unlikely that the observed effect is caused by a change in other medical therapies. The residual risk of AEs during the on-flecainide period may, to an unknown degree, be explained by proarrhythmic effects of flecainide, although this risk is presumably very low in these young patients without structural heart disease and coronary artery disease. It is conceivable that persisting significant ventricular ectopy (eg, frequent bigeminal ventricular premature beats, couplets, or nonsustained ventricular tachycardia) on the exercise stress test may identify the patients who are insufficiently protected by combination therapy with a beta-blocker and flecainide.

### Mechanism of Action

Throughout the years, flecainide’s mechanism of action has been debated extensively. The multiplicity of the drug’s actions has enabled different hypotheses about its effect of reducing arrhythmogenicity in CPVT.^24^ One hypothesis centers on a direct blocking effect on the open RyR2 channel, thereby limiting sarcoplasmic reticulum calcium (Ca^2+^) release through RyR2, and thus attenuating delayed afterdepolarizations and triggered activity.^20,25,26^ Another hypothesis revolves on its sodium channel blocking effects, which would reduce triggered activity,^27^ or prevent elevated intracellular Ca^2+^ levels by the Na^+^/Ca^2+^ exchanger.^28–30^ More recently, the former hypothesis was rendered more plausible, as Kryshtal et al showed that flecainide suppresses RyR2-mediated pathophysiological Ca^2+^ release even when cardiac sodium channels are completely inhibited.^31^ In addition, Kryshtal et al constructed a flecainide analogue that retains its sodium channel blocking properties, but has a much lower effect on the RyR2 single channels, which failed to inhibit RyR2-mediated sarcoplasmic reticulum Ca^2+^ release both in vitro and in vivo, in contrast with flecainide itself. The greater protection we observed in the more severely affected patients may be explained by studies demonstrating that increasing RyR2 activity increases the potency of flecainide, so-called “use-dependence.”^32^

### Clinical Implications

Current guidelines recommend adding flecainide to the medical therapy in (1) patients who have been resuscitated from SCA (class I), (2) patients who remain symptomatic on beta-blockers therapy (class IIa), and (3) patients in whom the suppression of the exertional VAs is incomplete (class IIa).^2^ Previous studies have shown that being symptomatic before diagnosis is an independent predictor of AEs in patients treated with beta-blockers.^3^ In our study, the most pronounced effect of flecainide was observed in patients who were previously symptomatic. Therefore, a treating physician may consider going directly to combination drug therapy with both nonselective beta-blocker and flecainide in all previously symptomatic patients rather than only in those with recurrent syncope or persistent exertional VAs on beta-blocker therapy.

### Strengths and Limitations of the Study

The case cross-over design of this study is a strength, eliminating potential confounding factors that may be present in a retrospective observational design. Another major strength of the present study is the large sample size. It is important that this allowed us to study the efficacy of flecainide with regard to AEs, rather than surrogate end points.

Although we performed extensive data checks to retrieve missing data, not all data were available for all patients because of the retrospective nature of the study. We cannot exclude the possibility of a selection bias. In addition, inherent to the study design, patients who died before the initiation of flecainide could not be included in the study, resulting in immortality-time bias. On the other hand, flecainide is most often prescribed in the highest-risk patients who have persistent VAs or in those who remain symptomatic on beta-blocker therapy, which may lead to confounding by indication.

Although beta-blocker type (selective or nonselective) was equal during the study periods, beta-blocker dose was nonequivalent in a small proportion of patients, which may have influenced outcomes. However, more than half of these patients had a lower beta-blocker dose during the on-flecainide period as compared with the pre-flecainide period, rendering it unlikely that this disparity led to an overestimation of the efficacy of flecainide.

In the primary analyses, we excluded patients who received an ICD at the time flecainide was initiated. If we had included these patients, 2 of the 4 components of the primary outcome (SCD and appropriate ICD shock) could have occurred only in the on-flecainide period. Previous studies have suggested that VAs that lead to an appropriate ICD shock do not always lead to syncope, SCA, or SCD in a patient without an ICD.^19^ Therefore, we believe that the inclusion of patients who received an ICD at the time flecainide was initiated would have resulted in an inappropriate comparison of AE rates in the pre-flecainide versus on-flecainide periods. However, because ICDs do not suppress VAs unlike beta-blockers, flecainide, or LCSD, we performed secondary analyses in which insertion of an ICD was not considered a change in background therapy (Supplemental Results). The results were very similar to the primary analyses.

### Conclusions

The present study provides evidence that flecainide use is associated with a significantly lower incidence of AEs when added to beta-blockers in patients with CPVT and particularly in patients with breakthrough AEs on beta-blocker therapy, further cementing its important role in the treatment of patients with CPVT.

## ARTICLE INFORMATION

### Acknowledgments

We acknowledge the valuable contribution of all International CPVT Registry and International Pediatric CPVT Registry collaborators.

### Sources of Funding

A.A.W. was funded by Predict-2, EU E-rare grant (Transnational Research Projects on Rare Diseases 2015, Improving CPVT). S.S. was funded by the Heart and Stroke Foundation (grant G-19-0024239). M.J.A. was supported by the Mayo Clinic Windland Smith Rice Comprehensive Sudden Cardiac Death Program. A.L. was funded by a grant from Programme Hospitalier de Recherche Clinique–PHRC 2014 (Ministère de la Santé N° AOR 04070). C.S. is the recipient of a National Health and Medical Research Council Practitioner Fellowship (No.1154992) and was supported by a New South Wales Health Cardiovascular Disease Clinician Scientist Grant. S.O. was funded by AMED (JP18ek0109202] and Grants-in-Aid for Scientific Research from the Japan Society for the Promotion of Science (15K09689). J.T.H. was funded by the John and Birthe Meyer Family Foundation. K.H.H. was funded by the Norwegian Research Council (ProCardio No.309762, GENE POSITIVE No. 288438, and EMPATHY No. 298736). E.Z. was funded by Mécanismes Proarythmiques Dépendant du Sodium et du Calcium, Agènce Nationale de la Recherche (ANR-19-CE14-0031-001).

### Disclosures

A.W. is a consultant for ARMGO and LQT Therapeutics. S.S. is a consultant for Cardurion. M.J.A. is a consultant for Abbott, Boston Scientific, Bristol Myers Squibb, Daiichi Sankyo, Invitae, Medtronic, Tenaya Therapeutics, and UpToDate. M.J.A. and the Mayo Clinic are involved in an equity/royalty relationship with AliveCor, Anumana, Thryv Therapeutics, and Pfizer. However, none of these entities were involved in this study. S.P.E. is a consultant for UptoDate and has a relationship with Pfizer. S.C.Y. is a consultant for Boston Scientific and has received research grants from Medtronic and Biotronik. The other authors report no conflicts.

### Supplemental Material

Tables S1–S3

Expanded Results

## Supplementary Material
