# Supplementary material for: Flecainide Is Associated With a Lower Incidence of Arrhythmic Events in a Large Cohort of Patients With Catecholaminergic Polymorphic Ventricular Tachycardia
Source: Circulation. 2023 Oct 27;148(25):2029–37. doi: 10.1161/CIRCULATIONAHA.123.064786 (PMC10727202; doi:10.1161/CIRCULATIONAHA.123.064786)
Supplement: Supplementary file 1 [file cir-148-2029-s001.pdf]

Supplemental material

Supplementary results

Analysis including the patients who had an ICD implanted within 7 days of flecainide initiation

The first analysis included the patients who were excluded because they received an ICD within 7 days of flecainide initiation. In this analysis including 251 patients, the implantation of an ICD during any study period was still a cut-off point for determining follow-up duration as it was in our main analysis. Thus, if a patient received an ICD while being treated with beta-blocker monotherapy, the ICD implantation date was the start of the pre-flecainide period. If a patient received an ICD while being treated with a beta-blocker and flecainide, the ICD implantation date was the end of the on-flecainide period. In this analysis, 4 additional patients could be included (n=251). Three of the newly included patients had no arrhythmic events during both study periods. One patient had one arrhythmic event during the pre-flecainide period and none during the on-flecainide period. Using generalized linear mixed model (GLMM) with the assumption of a negative binomial distribution, there was a significant reduction in the rate of arrhythmic events after flecainide (IRR 0.56 [0.36-0.86], p=0.009).

Analysis including the patients who had an ICD implanted within 7 days of flecainide initiation *and* where the date of ICD implantation was not a cutoff point in limiting follow-up duration.

The second supplementary analysis included the patients who were excluded because they received an ICD within 7 days of flecainide initiation *and* the implantation of an ICD during any study period was *not* a cutoff point for determining follow-up duration in this analysis. In this analysis, 251 patients were included. The median pre-flecainide follow-up duration was 2.9 years (IQR 0.6-7.6) and the median on-flecainide follow-up duration was 3.5 years (IQR 1.5-6.9). During the pre-flecainide period, 45 patients experienced 67 arrhythmic events. During the on-flecainide period, 29 patients experienced a total of 51 arrhythmic events. Using negative binomial GLMM, there was a significant reduction in the rate of arrhythmic events after flecainide (IRR 0.66 [0.45-0.96], p=0.028).

Characteristics of cases excluded for unequal background therapy

A total of 142 patients were excluded based on not being on equal background therapy for at least 7 days pre and/or post-flecainide. Of these, 107 (75%) were probands. Their median age at diagnosis was 14 years (IQR 9-22). Twenty-six (18%) patients were asymptomatic before diagnosis. Thirty-nine (27%) had arrhythmic events after diagnosis. The indications for starting flecainide were CPVT-related symptoms (n=15), ventricular arrhythmia or ventricular tachycardia despite other medication

(n=66), initial therapy (n=38), patient preference (n=4), other (n=15), or unknown (n=4). In conclusion, these patients were slightly younger and more symptomatic before diagnosis and on initial therapy as compared with the study population.

Supplementary tables

Table S1. Commonly used β-blockers, their dosing and frequency regimen, and indications. Reproduced from T.M. Roston et al.[18]

β-blocker	Route	Frequency	Starting dose, mg/kg/d	Typical adult starting dose	Maximum dose, mg/kg/d	Typical adult maximal dose	Equivalent daily dose	Specific cardiac indications	Common noncardiac indications	Additional considerations
Acebutolol	PO	OD or BID	1	200-400 mg divided OD or BID	20	600 mg BID	200 mg/d	HTN,* stable CAD	Hyperthyroidism (although other agents preferred)	> 100 mg/d unlikely to offer further antihypertensive benefit; higher doses often needed for angina
Atenolol	PO	OD or BID	0.5	25-50 mg OD	2	200 mg OD	50 mg/d	HTN,* MI, stable CAD	Hyperthyroidism (second-line)	Not recommended in pregnancy > 100 mg/d unlikely to offer further antihypertensive benefit
Bisoprolol	PO	OD	0.04	1.25-2.5 mg OD	0.14	20 mg OD	5 mg/d	HFrEF, arrhythmia, HTN (second-line)*		May use up to 20 mg OD for hypertension; Target dose for HFrEF: 10 mg OD
Carvedilol	PO	BID	0.1	3.125 mg BID	0.7	50 mg BID	25 mg/d	HFrEF, HTN (second-line),* MI with EF < 40%	Esophageal varices (second-line)	Target dose in HFrEF: 50 mg BID
Labetalol	PO	BID (for large doses may divide TID or QID)	3	100 mg BID	10	800 mg TID	200 mg/d	HTN in pregnancy, HTN crisis,	Subarachnoid hemorrhage	Safe in pregnancy
Metoprolol	PO/I.V.	Depends on formulation	1	12.5-25 mg BID	6 (up to 400 mg/d)	200 mg BID	100 mg/d 50 mg BID	HTN,* HFrEF, arrhythmia, MI, stable CAD	Migraine (second-line), hyperthyroidism (second-line)	Safe in pregnancy, only succinate formulation

										indicated in HFrEF
Nadolol	PO	OD	0.6	40 mg OD	3.4	320 mg OD	80 mg/d	HTN,* CAD, arrhythmia, long QT syndrome	Esophageal varices; migraine	
Nebivolol	PO	OD	0.07	5 mg OD	0.6	40 mg OD	10 mg/d	HFrEF, HTN (second-line)		Useful in severe bronchospasm
Propranolol	PO/I.V.	Depends on formulation	1	40 mg BID	5	40 mg QID (arrhythmia) or 80 mg QID (angina)	80 mg/d	Arrhythmia, long QT syndrome, HTN,* obstructive hypertrophic cardiomyopathy, stable CAD, MI	Hyperthyroidism; antipsychotic-induced akathisia (second-line), essential tremor, migraine, performance anxiety, esophageal varices	Superior to metoprolol in arrhythmic storm, used for hyperthyroidism in pregnancy
Sotalol	PO	BID	1	40-80 mg BID	4	160 mg BID	80 mg/d	Arrhythmia, HOCM, ARVC		Useful in AF or VT with EF > 35%<br>Assess baseline QTc and creatinine before initiation; adjust dose for renal impairment
Esmolol	I.V.	Continuous infusion	1 (bolus); 0.05 infusion	500 μg/kg load, then 50 μg/kg/min infusion	0.2	200 μg/kg/min	N/A	Intraoperative arrhythmia or arrhythmia in shock	Hyperthyroidism	Rapid onset and offset are ideal in the critical care setting

Equivalent daily dose is on the basis of an approximate conversion between the commonly prescribed doses for each type of β-blocker. Data are derived from Lexicomp.com, PrescribersLetter.com, BC Children's & Women's Hospital Pharmacy Formulary, Vancouver General Hospital Pharmacy Formulary, and Kaiser Permanente Formulary.

AF, atrial fibrillation; ARVC, arrhythmogenic right ventricular cardiomyopathy; BID, twice daily; CAD, coronary artery disease; EF, ejection fraction; HFrEF, heart failure with reduced ejection fraction; HOCM, hypertrophic obstructive cardiomyopathy; HTN, hypertension; I.V., intravenous; MI, myocardial infarction; N/A, not applicable; OD, once daily; PO, by mouth; QID, 4 times daily; TID, 3 times daily; VT, ventricular tachycardia.

* Although certain β-blockers are approved as first- or second-line antihypertensive agents, contemporary guidelines do not support the use of β-blockers as a first-line option for primary HTN.

Table S2. Characteristics of patients not treated with flecainide

	Patients not treated with flecainide (n=944)	Study cohort (n=247)
**Year of enrolment in registry, median (IQR)**	2017 (2015-2017)	2016 (2014-2017)
**Female sex, n (%)**	507 (54)	123 (50)
**Proband, n (%)**	326 (35)	156 (63)
**Asymptomatic before diagnosis, n (%)**	452 (48)	83 (34)
**Sudden cardiac arrest before diagnosis, n (%)**	72 (8)	48 (19)
**Age at diagnosis, median (IQR)**	19 (11-43)	13 (9-22)
**Beta-blocker therapy, n (%)**	760 (81)	247 (100)
**Intentional non-therapy, n (%)**	168 (18)	0 (0)
**ICD, n (%)**	203 (22)	70 (28)*
**LCSD, n (%)**	37 (4)	21 (9)*
**Arrhythmic event after diagnosis, n (%)**	110 (12)	59 (24)

*ICDs implanted and LCSDs performed at baseline. ICD, implantable cardioverter-defibrillator; LCSD, left cardiac sympathetic denervation.

Table S3. Characteristics of patients with arrhythmic events during the on-flecainide period

	All patients (n=23)	Suboptimal* therapy (n=7)	Optimal therapy (n=16)
**Female, n (%)**	10 (43)	2 (29)	8 (50)
**Mental retardation/neurologic disease, n (%)**	9 (39)	1 (14)	8 (50)
**Genotype unknown/not evaluated, n (%)**	2 (9)	0	2 (13)
**Genotype negative, n (%)**	1 (4)	0	1 (6)
**Genotype positive**			
***RYR2*, n (%)**	19 (83)	7 (100)	12 (75)
***CASQ2*, n (%)**	1 (4)	0	1 (6)
**Worst symptom prior to diagnosis**			
**Asymptomatic, n (%)**	4 (17)	2 (29)	2 (13)
**Syncope, n (%)**	11 (43)	4 (57)	7 (44)
**Sudden cardiac arrest, n (%)**	8 (35)	1 (14)	7 (44)
**Age at symptom onset, years, median (IQR)**	10 (6-14)	12 (11-14)	9 (6-13)
**Age at diagnosis, years, median (IQR)**	11 (8-13)	11 (8-13)	11 (8-15)
**Age at flecainide initiation, years, median (IQR)**	13 (11-18)	14 (11-22)	13 (11-17)
**Age at first breakthrough event, years, median (IQR)**	16 (13-20)	15 (14-22)	16 (13-20)

*Flecainide therapy was considered suboptimal when patient was non-adherent or was prescribed a low flecainide dose (<100 mg). Patients in whom information on adherence was missing were classified as adherent. Patients who experienced at least 1 arrhythmic event on an optimal dosage of flecainide and in whom non-adherence was not confirmed were classified as having optimal therapy.
